# A Challenging Diagnosis of IgG4-Related Disease When Understanding Limitations of Laboratory Testing Was Pivotal

**DOI:** 10.1155/2017/8748696

**Published:** 2017-07-30

**Authors:** Victoria Y. Y. Xu, Mary Bell, Alireza Zahirieh, Janey Hsiao, Kevin Higgins, Zeina Ghorab, Arthur Bookman, Pak Cheung Chan

**Affiliations:** ^1^Department of Medicine, University of Toronto, Toronto, ON, Canada; ^2^Division of Rheumatology, Sunnybrook Health Sciences Centre, Toronto, ON, Canada; ^3^Division of Nephrology, Sunnybrook Health Sciences Centre, Toronto, ON, Canada; ^4^Division of Hematology/Medical Oncology, Sunnybrook Health Sciences Centre, Toronto, ON, Canada; ^5^Department of Otolaryngology/Head and Neck Surgery, University of Toronto, Toronto, ON, Canada; ^6^Department of Anatomic Pathology, Sunnybrook Health Sciences Centre, Toronto, ON, Canada; ^7^Department of Laboratory Medicine and Pathobiology, University of Toronto, Toronto, ON, Canada; ^8^Multi-Disciplinary Sjogren's Clinic, University Health Network, Toronto, ON, Canada; ^9^Department of Clinical Pathology, Sunnybrook Health Sciences Centre, Toronto, ON, Canada

## Abstract

A 76-year-old man was incidentally found on a CT scan to have lymphadenopathy and bilateral kidney enlargement suggestive of infiltrative renal disease. He was largely asymptomatic but had bilateral salivary and lacrimal gland enlargement. A grossly elevated serum IgG (>70 g/L) with concomitant suppression of other immunoglobulins, a small IgG restriction, and a parotid biopsy revealing lymphoplasmacytic infiltrate with slight kappa light chain excess all suggested a lymphoproliferative disorder (LPD). The diagnostic workup was further confounded by a normal serum IgG4 concentration. Moreover, bone marrow and renal biopsies did not reveal evidence of LPD. Discussion with the laboratory not only clarified that the markedly increased total IgG could not be accounted for by the small IgG restriction, but also identified a discrepancy in the IgG4 measurement. Repeat analysis of a follow-up sample revealed an elevated IgG4 of 5.94 (reference interval: 0.039–0.864) g/L, which prompted a repeat parotid biopsy that showed predominant IgG4+ lymphocytic infiltrates. Despite the deluding presentations, a final diagnosis of IgG4-related disease (IgG4-RD) was made based on elevated serum IgG4 concentrations and histopathological findings. This case highlights the importance of recognizing limitations of laboratory testing and the benefit of close communications among clinical subspecialties and the laboratory.

## 1. Introduction

Immunoglobulin G4-related disease (IgG4-RD) is an immune-mediated multisystem disease that is characterized by inflammation and fibrosis of affected organs [1, 2]. Since the disease often presents with nonspecific symptoms and signs and can affect any organ or system, the diagnosis is challenging and often delayed. The diagnosis of IgG4-RD requires the integration of clinical, laboratory, and histopathologic information.

We report a case of IgG4-RD diagnosed in a patient who was incidentally found to have lymphadenopathy on a routine CT scan for evaluation of aortic aneurysm repair. His manifestations of IgG4-RD included bilateral painless lacrimal, submandibular and parotid gland enlargement (Mikulicz features), infiltrative renal disease with moderate renal dysfunction, and possibly aortitis leading to further aneurysm extension after repair. The diagnosis was initially confounded by laboratory results that were inconclusive or misleading (a very high total IgG of >70 g/L with detection of a monoclonal gammopathy along with a falsely normal serum IgG4 level). Recognition of the limitations of laboratory testing and correlation of clinical findings with immunohistochemistry ultimately confirmed the diagnosis of IgG4-RD.

## 2. Case Presentation

A 76-year-old man was referred by his vascular surgeon to Rheumatology for query connective tissue disease after he was incidentally found to have increased lymphadenopathy and bilateral enlarged kidneys on follow-up CT imaging after thoracic endovascular aortic repair (TEVAR).

His surgical history was significant for abdominal aortic aneurysm repaired with stent and type B dissection of thoracoabdominal aortic aneurysm with TEVAR repair three years later. His medical history was significant for hypertension, dyslipidemia, and sinusitis. He had no known drug allergies. His medications included aspirin, ramipril, metoprolol, and atorvastatin.

Further history indicated that he had experienced progressive painless swelling in his bilateral cheeks/neck for five years. On review of systems, he also endorsed intermittent sinusitis, dry mouth, multiple dental cavities, and Raynaud's phenomenon. He denied any constitutional symptoms.

On examination, he had nontender bilateral parotid gland enlargement and bilateral lacrimal gland hypertrophy. He had chronic perforation of his right tympanic membrane and fragmented decayed dentition. The rest of the examination was unremarkable.

Workup for connective tissue disease, including ANA, ENA, ANCA, HIV, hepatitis B and C serology, ESR, CRP, and cryoglobulin screen, was all negative. There was markedly elevated IgG (76.71 g/L) with concomitant suppression of IgA and IgM levels. Serum protein electrophoresis (SPE) suggested an M-protein band of 68 g/L in the beta gamma interzone. Serum immunofixation electrophoresis (IFE), however, identified only a small clonal restriction in IgG while urine IFE identified a very tiny kappa restriction. Serum free light chain assays showed elevated free kappa and lambda concentrations (both >300 mg/L) with a normal ratio. Blood film showed rouleaux, normochromic anemia, mild eosinophilia, few atypical lymphocytes, and slight thrombocytopenia. Chest X-ray showed no hilar adenopathy. Sinus X-ray showed opacification of paranasal sinuses possibly from inflammatory diseases. Initial submandibular gland and lymph node biopsy was nondiagnostic and revealed lymphoplasmacytic infiltrate with slight kappa light chain excess.

**Figure 1:**
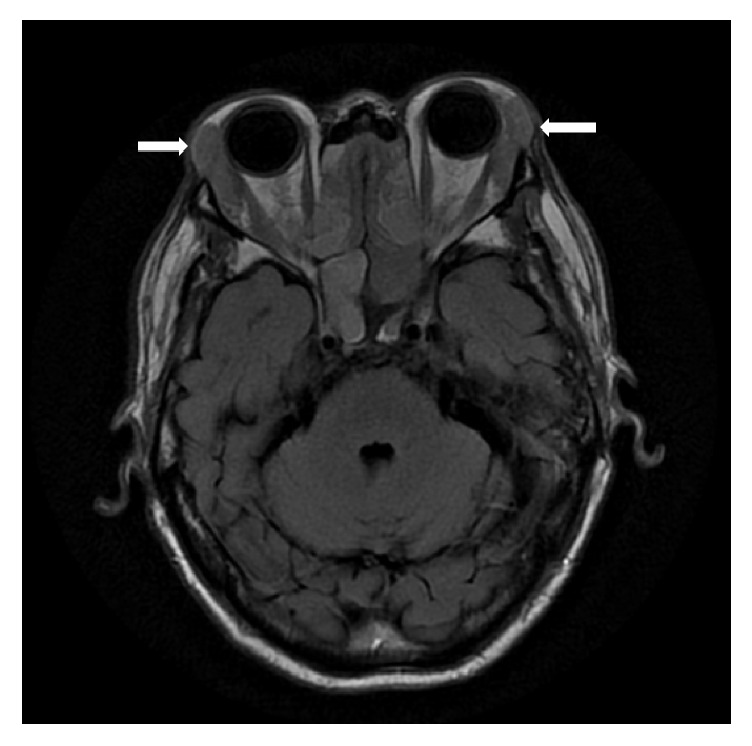
MRI brain showing bilateral enlargement (arrows) of the lacrimal glands.

He was referred to Haematology, as the grossly elevated serum IgG (>70 g/L) with concomitant suppression of other immunoglobulins, small IgG restriction, and biopsy results raised concern for LPD. Imaging for lymphoma protocol was performed. CT scan of the abdomen and pelvis showed multiple lymph nodes and bilateral kidney enlargement with infiltrative and nodular disease. There was also circumferential periaortic wall thickening. MRI brain showed bilateral enlargement of parotid and lacrimal glands as well as chronic sinusitis (see Figure 1).

Further discussion with the laboratory revealed that the relative contribution of M-protein was most likely overestimated, since SPE has limited ability to distinguish clonal from polyclonal expansion, especially in the setting of very high total IgG as in this case. Serum and urine IFE were in line with a minor monoclonal gammopathy but were not in keeping with such a marked IgG increase, and the normal free light chain ratio was inconsistent with a large clonal process. Bone marrow biopsy and aspirate showed no evidence of amyloidosis or plasma cell, lymphoproliferative, or myeloproliferative disorder. The patient may have a coincidental monoclonal gammopathy of undetermined significance (MGUS) which may or may not be the result of a reactive immunologic process.

Given the imaging findings and renal dysfunction (eGFR = 39 mL/min/1.73 m^2^), the patient was also referred to Nephrology for a kidney biopsy which showed atypical interstitial lymphoid infiltrate with some eosinophils and occasional plasma cells but was negative for IgG, IgM, IgA, C3, C1q, lambda, and kappa light chains by immunofluorescence.

Initial serum IgG4 level was reported as normal by an outside referral lab (Lab A). However, the sum of IgG subclasses did not equate to the total IgG concentration reported, and further consultation with a Biochemist led to measurement at a different lab (Lab B) but the result was discrepant (see Table 1). On further repeat testing, Lab A again underestimated the IgG4 level, while an outside consulting lab (Lab C) confirmed an elevated IgG4 level of 5.94 g/L which was in line with earlier findings from Lab B.

**Figure 2:**
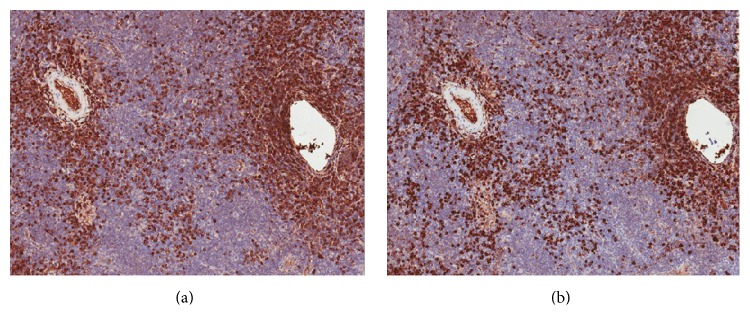
Immunostaining for IgG (a) and IgG4 (b) from submandibular gland/lymph node biopsy.

**Figure 3:**
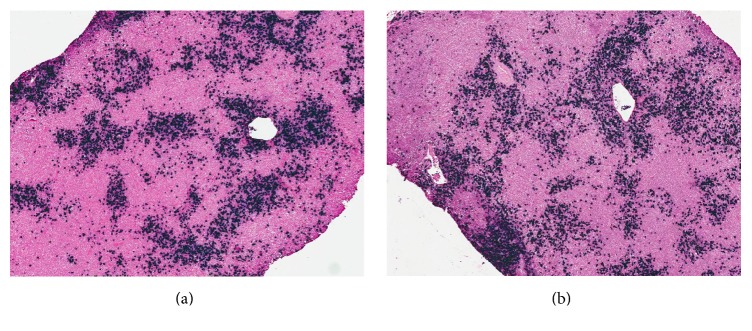
Kappa (a) and lambda (b) mRNA by in situ hybridization (showing polytypic plasma cells) from submandibular gland/lymph node biopsy.

The elevated IgG4 level prompted a repeat submandibular excisional biopsy. On this tissue sample, flow cytometry indicated no clonal process and was not supportive of lymphoproliferative disorder. Further, molecular diagnostic studies by PCR did not reveal any evidence of B-cell clonal restriction. However, immunohistochemistry studies showed IgG4 positive plasma cells present in increased numbers (clusters of >100) with IgG4/IgG ratio of >40% (see Figure 2). Furthermore, in situ hybridization for kappa and lambda light chain mRNA confirmed that these plasma cells were polytypic (see Figure 3). While there was a lack of characteristic “storiform” fibrosis on biopsy, this histopathologic feature is reportedly uncommon in tissues such as lymph nodes and salivary glands [3].

A final diagnosis of IgG4-RD was made based on clinical manifestations, elevated IgG4, and histopathologic findings.

Immunosuppressive treatment was deferred as the patient was awaiting an extensive dental procedure. He had close follow-ups with Rheumatology and Nephrology. Nearly one year after his diagnosis, he showed significant reductions of his glandular enlargement and stable kidney function. His total IgG dropped from 77 to ~24 g/L while his IgG4 level remained elevated at 6 g/L. Spontaneous resolution of clinical manifestations has been described in IgG4-RD patients who received no treatment. A recent systematic review examining therapeutic approaches to IgG4-RD reported that no therapy (wait-and-see management) was used in 13% of all patients included (264 out of 1952). There were a greater percentage of patients treated without any therapeutic intervention in specific organ involvement: 71% and 35% of those with lymphadenopathy and salivary gland involvement, respectively. Spontaneous resolution was seen in 43% (68 of 159) of patients managed without therapy, although there were higher relapse rates compared to those treated with glucocorticoids. The review concludes that wait-and-see management may be appropriate in asymptomatic patients with lymphadenopathy or mild salivary gland enlargement [4]. The patient in this case continues to be followed regularly by Rheumatology and Nephrology.

## 3. Discussion

After an extensive workup to exclude LPD and malignancy, the patient was diagnosed with IgG4-RD affecting his salivary glands, lacrimal glands, and likely both kidneys. The presence of bilateral renal enlargement with infiltrative hypoattenuating regions provided strong radiographic support for IgG4-related renal disease. The renal biopsy yielded only four glomeruli but demonstrated the presence of an interstitial lymphoid infiltrate with occasional plasma cells. Immunofluorescence was negative for IgG, IgA, IgM, C3, C1q, kappa, and lambda. The pathological features of IgG4-related renal disease can include tubulointerstitial nephritis (TIN) with dense lymphoplasmacytic infiltration, storiform fibrosis, and IgG4-positive plasma cell infiltration [5]. Additionally, membranous nephropathy is a rare pathologic finding. However, as IgG4-RD can be patchy, renal biopsies can on occasion reveal normal histology [5]. Although no tissue from his aortic aneurysm repair was sent for pathology, his CT chest showed periaortic wall thickening as can be seen in IgG4-related aortitis [2].

A clinically significant problem highlighted by this case was the lab-to-lab variability in serum IgG4 measurements and the diagnostic utility of IgG4 levels in IgG4-RD. An IgG4 level of 1.35 g/L evaluated in the diagnosis of autoimmune pancreatitis has been widely accepted as the cut-off level for IgG4-RD [6, 7]. In fact, serum IgG4 levels were first thought to be a crucial part of the diagnosis of IgG4-RD, but recent evidence has revealed significant limitations [8]. Serum IgG4 levels are elevated in as many as 84% of patients with IgG4-RD [8, 9]. However, approximately 30% of patients with classic histological and immunohistochemical findings have normal serum IgG4 levels [1]. A recent North American study examining test characteristics of serum IgG4 concentrations for the diagnosis of IgG4-RD in patients with multiorgan disease found a sensitivity of 90% and negative predictive value of 96%, but poor specificity (60%) and poor positive predictive value (34%) [9]. However, higher serum IgG4 levels have been correlated with multiple organ involvement [1]. Current research has focused on biomarker discovery using technologies such as flow-cytometry-based assays [10]. Whether these assays are more reliable than serum IgG4 levels for diagnosis and assessment of disease activity remain to be seen.

It is important for clinicians to be aware of the limitations of laboratory tests. In this case, the overestimated M-protein in the SPE result and falsely normal initial serum IgG4 level created a diagnostic conundrum and led to possible delays in diagnosis. One likely explanation for the initial falsely negative IgG4 level stems from intrinsic properties of the IgG4 immunoassay, specifically the prozone effect. This phenomenon can occur in the setting of antigen excess (in this case, very high serum IgG4 concentrations), where assays do not provide accurate IgG4 measurements if the sample has not been diluted appropriately [9, 11]. The prozone effect has been described in another case of IgG4-RD, with an initially normal serum IgG4 level that was found to be elevated after dilution of the sample [12]. The UK National External Quality Assessment Schemes (UK NEQAS) and other publications have shown that the antigen excess effect can occur at levels of IgG4 around the 95th centile of a normal population, causing missed IgG4 elevations due to prozoning [13]. Assays were traditionally designed to detect low to normal IgG4 levels in order to diagnose immune deficiency; however, the recent recognition of IgG4-RD as a disease entity calls for a change in the laboratory test methods to better detect elevated IgG4 levels [13]. A new protocol with a two-step check has been proposed to minimize the prozone effect and should be implemented in all laboratories measuring IgG4 [13].

The discrepancies in laboratory measurements in this case were acknowledged and discussed among the various specialists involved, including the Haematologist, Biochemist, and Pathologist, highlighting the importance of open communication among care providers in the face of diagnostic dilemmas.

## Figures and Tables

**Table 1:** IgG subclass analysis for this patient showing varying values at different labs.

Sample date	September 2015		February 2016		
Performing lab	Lab A	Lab B	Lab A	Lab C	Reference intervals (g/L)
IgG1 (g/L)	13.8	18	19.1	20.0	3.82–9.29
IgG2 (g/L)	5.64	>10.1	46.13	43.20	2.42–7.00
IgG3 (g/L)	1.36	>2.1	1.01	6.69	0.22–1.76
IgG4 (g/L)	0.734^*∗*^	>3.3	0.309^*∗*^	5.94	0.039–0.864
Calculated total IgG (g/L)	21.5		66. 6	75.8	

Reported total IgG by home lab (g/L)	69.5		76.7		6.1–16.2

^*∗*^IgG4 levels were underestimated at Lab A.

## Additional Points

(i) IgG4-related disease (IgG4-RD) is a systemic condition that often affects multiple systems, resulting in a wide variety of clinical manifestations. (ii) IgG4-RD can clinically and histopathologically mimic many conditions including lymphoproliferative diseases; biopsy should be obtained to confirm IgG4-RD and exclude malignancy. (iii) Serum IgG4 level alone should not be relied upon for diagnosis of IgG4-RD, which requires integration of clinical, laboratory, and histopathological findings. (iv) When serum IgG4 measurement appears discordant with the clinical suspicion of disease activity, consider the limitations of laboratory tests such as prozone effect leading to falsely normal IgG4 concentrations. (v) Collaboration and communication among specialists, including the lab, are essential in solving diagnostic conundrums.
